# Mithramycin and Analogs for Overcoming Cisplatin Resistance in Ovarian Cancer

**DOI:** 10.3390/biomedicines9010070

**Published:** 2021-01-12

**Authors:** David Schweer, J. Robert McCorkle, Jurgen Rohr, Oleg V. Tsodikov, Frederick Ueland, Jill Kolesar

**Affiliations:** 1 Department of Obstetrics and Gynecology, Division of Gynecologic Oncology Lexington, University of Kentucky Markey Cancer Center, Lexington, KY 40536, USA; dssc224@uky.edu (D.S.); fuela0@uky.edu (F.U.); 2 Department of Pharmaceutical Sciences, College of Pharmacy, University of Kentucky, Lexington, KY 40536, USA; rob.mccorkle@uky.edu (J.R.M.); jurgen.rohr@uky.edu (J.R.); oleg.tsodikov@uky.edu (O.V.T.)

**Keywords:** ovarian cancer, mithramycin, Sp1, novel therapeutics, platinum-resistant

## Abstract

Ovarian cancer is a highly deadly malignancy in which recurrence is considered incurable. Resistance to platinum-based chemotherapy bodes a particularly abysmal prognosis, underscoring the need for novel therapeutic agents and strategies. The use of mithramycin, an antineoplastic antibiotic, has been previously limited by its narrow therapeutic window. Recent advances in semisynthetic methods have led to mithramycin analogs with improved pharmacological profiles. Mithramycin inhibits the activity of the transcription factor Sp1, which is closely linked with ovarian tumorigenesis and platinum-resistance. This article summarizes recent clinical developments related to mithramycin and postulates a role for the use of mithramycin, or its analog, in the treatment of platinum-resistant ovarian cancer.

## 1. Introduction

Ovarian cancer is the fifth deadliest malignancy in US women. In 2020, an estimated 21,750 new cases and approximately 13,940 deaths related to ovarian cancer were predicted [1]. There are no uniformly accepted screening tests for ovarian cancer. Early disease symptoms are often vague and non-specific, including pelvic discomfort, bloating, changes in gastrointestinal or genitourinary habits. For this reason, more than 75% of new cases are diagnosed at stage III or greater. Epithelial histopathology accounts for approximately 90% of malignant ovarian neoplasms (90%) [2]. The mainstay of advanced-stage ovarian cancer treatment is typically a combination of surgery (staging and debulking) and systemic chemotherapy with a taxane-platinum doublet [3]. Despite a high likelihood of clinical response from primary therapy, more than 80% of advanced stage disease will recur [4].

Recurrent ovarian cancer is considered “incurable”. A specific prognosis is dependent upon sensitivity to platinum-based agents. There is an exceedingly poor prognosis in platinum-resistant disease, with an estimated overall survival of 9–12 months [5]. Typical salvage regimens utilize sequential single-agents, as multiagent regimens have not provided benefits but only increased toxicity [6,7,8]. In the setting of platinum-resistant disease, single-agent chemotherapy response rates are <20% with an expected progression free survival of only 3-4 months. Due to dismal survival outcomes, there is a demonstrated need for new or modified therapies in the setting of platinum-resistant ovarian cancer. Improved understanding of the mechanisms of platinum resistance may help identify new and effective treatment strategies for this patient population [4,5].

The primary mechanism of action of platinum compounds is covalent binding of platinum to the N7 atoms of guanine bases of DNA with the creation of monoadducts and intra- and/or interstrand cross-links. These DNA lesions, of which interstrand crosslinks (ICL) are the most toxic, act as replication and transcription roadblocks that inhibit cell division and ultimately trigger cell death [9,10]. There are several mechanisms associated with platinum resistance, including increased drug efflux, enhanced DNA repair, and intracellular protein alterations that can sequester or modify platinum agents [11,12]. Enhanced DNA repair is considered a key driver of platinum-resistance. One of several DNA repair mechanisms is engaged depending on the nature of the crosslink, such as base excision repair (BER), mismatch repair (MMR), homologous recombination (HR), and nucleotide excision repair (NER) [11,13]. The NER pathway is the repair pathway responsible for the removal of platinum monoadducts and intrastrand crosslinks. NER involves ~20 proteins, culminating with incisions of the damaged DNA strand on both sides of the lesion by the XPG endonucleases and the heterodimer XPF-ERCC1 [14]. Since XPF-ERCC1 is also involved in ICL repair, this endonuclease is especially critical [15,16].

Other mechanisms of resistance deal with the influx and efflux of platinum drugs in target cells. The intracellular concentration of platinum compounds determines the number of DNA lesions produced by the drug [17]. Copper transporter-1 (CTR1) transports platinum into the cell, and its downregulation can reduce the intracellular accumulation of platinum compounds [18]. Multi-drug transporters (e.g., MDR1) are involved in the active efflux of platinum-agents [5]. Additionally, glutathione, an intracellular tripeptide, has been associated with platinum drug metabolism and interference with platinum-induced DNA damage [5,11].

Novel therapeutics that can overcome platinum-resistance could significantly impact ovarian cancer mortality. With this goal in mind, the identification of novel therapeutics for the treatment of platinum-resistant ovarian cancer is of critical importance [19]. The purpose of this article is to review the role of mithramycin, an antineoplastic antibiotic, and its analogs as a potential therapeutic agent in overcoming platinum resistance in ovarian cancer. 

## 2. Mithramycin

**Figure 1:**
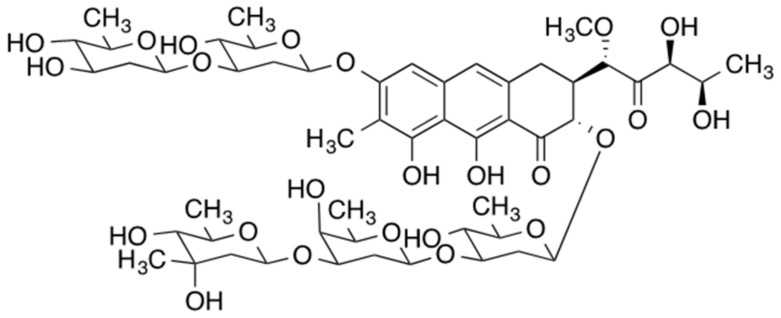
Chemical structure of Mithramycin [23].

Mithramycin, also referred to as plicamycin, is an antineoplastic antibiotic that is naturally produced by three bacteria of the *Streptomyces* genus (*S. argillaceus*, *S. tanashiensis*, and *S. plicatus*) (Figure 1) [20]. Historically, some leukemias and testicular cancer were treated with mithramycin, in addition to Paget’s disease and the associated hypercalcemia. Despite its anticancer activity, the use of mithramycin was limited due to its very narrow therapeutic window and severe toxicity, resulting in discontinued commercial production in 2000 [10]. The discovery of mithramycin’s activity against Ewing sarcoma, bone and soft tissue pediatric cancer in vitro and in mouse tumor xenografts have led to a phase I/II trial of mithramycin as a monotherapy against Ewing sarcoma [21,22]. This trial, too, was terminated due to the inability to reach pharmacologically relevant mithramycin levels in plasma before the onset of adverse effects.

## 3. Use of Mithramycin in Ovarian Cancer

The clinical use of mithramycin for ovarian cancers has largely been limited to the 1960s. In a case series studying the use of mithramycin in “embryonal cancers”, a form of germ cell tumors, utility in primary testicular tumors was noted; however, a metastatic ovarian teratoma was included in the series with marked remission [24]. A small clinical trial using mithramycin as salvage chemotherapy in 26 patients with advanced metastatic malignancy has also been reported. Two patients had ovarian pathology. While the majority (16/26) of patients did not have a therapeutic response, one ovarian subject had “temporary arrest” of the disease, while the other ovarian subject had quantitative remission. Regarding adverse side effects, they were few and relatively mild [25]. A follow-up clinical trial in 1968, involved 32 subjects with advanced malignancies of multiple primary tumors. There was a single ovarian cancer subject that had progression of disease on therapy. Interestingly, 10/32 subjects had no adverse cytotoxic effects from therapy. Reflecting the highly advanced nature of the subjects’ disease, 10/32 subjects died within two weeks of therapy [26].

## 4. Recent Clinical Development

Interest in mithramycin was renewed after a high-throughput assay-driven discovery of this molecule as an inhibitor of EWS-FLI1, an abnormal oncogenic fusion. EWS-FLI1 is an aberrant transcription factor causing Ewing sarcoma and is present only in tumor cells. Mithramycin demonstrated anti-tumor effects both in vitro and in vivo in Ewing sarcoma studies [22]. This led to a phase I/II clinical study of mithramycin monotherapy in Ewing sarcoma (NCT01610570). This was ceased due to hepatotoxicity [21,27]. Approximately 75% of treated patients had dose limited hepatotoxicity, with acute injury starting within days of administration. Hepatotoxicity was noted at 25 µg/kg/dose, requiring a dose de-escalation. As a consequence, no efficacy was demonstrated. Notable transaminitis was seen within 72 h of treatment, reaching levels up to 500× the upper limit of normal, with resolution often taking 1–3 weeks [28,29]. Recent investigations suggested that the hepatotoxicity was related to the inhibition of farnesoid-X receptor signaling and germline variants in genes involved in bile acid flow and disposition (e.g., ABCB4 and ABCB11) [29]. Interestingly, the associated hepatic injury was transient, rarely symptomatic, and did not require intervention; however, it significantly hampered the clinical utility of mithramycin [21,29].

## 5. Mechanism of Action

**Figure 2:**
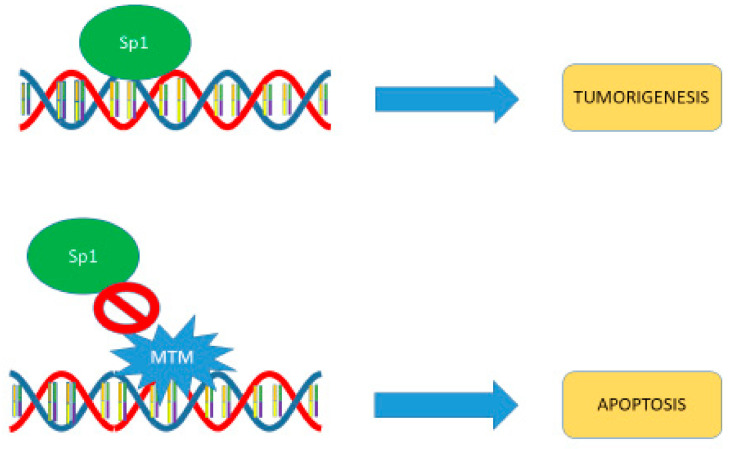
Mithramycin inhibits Sp1 binding to DNA, which may result in increased apoptosis rather than enhanced tumorigenesis.

The primary mechanism of action of mithramycin is recognized to be in its control of transcription via noncovalent binding of this molecule as a Mg^2+^-coordinated dimer to X(G/C)(G/C)X DNA sites in the minor groove (here, X is any base) [30,31,32,33,34,35,36]. Perturbing effects of mithramycin on transcription were observed for G/C-rich Sp1-regulated promoters [37,38,39,40,41]. This partially selective inhibition of promoters controlled by Sp1, thought to be relevant for ovarian cancer, is associated with a number of antineoplastic effects, including increased apoptosis, decreased angiogenesis, and inhibition of cell growth (Figure 2). Inhibition of Sp1-dependent transcription is associated with significant anticancer activity across multiple cell lines. [42]. Even though Sp1-controlled transcription has been viewed historically as a prominent target of mithramycin, ultimately, the effect of mithramycin on cancer cells is likely to be an indirect consequence of perturbing the function of Sp1 or some other transcription factor. Such indirect effects appear to be responsible for upregulation of p53 in malignant mesothelioma [43], as well as gynecologic cancers [44].

## 6. SP1

Specificity protein 1 (Sp1) is a zinc finger transcription factor first described in 1986 with an affinity for GC rich promoter regions [45,46,47,48]. Sp1 regulates multiple cellular functions and has been repeatedly linked to oncogenesis with its involvement in the expression of the cell cycle, angiogenesis, DNA damage and repair, and apoptosis genes. Upregulation of Sp1 expression is a poor prognostic factor in multiple cancer types, including gastric, pancreatic, and lung [42,49,50,51,52,53]. While Sp1 has been an enduring target for chemotherapy, transcription factors have traditionally been viewed as “undruggable”. The rationale stems from the lack of small hydrophobic cavities for compounds on the binding surface of transcription factors. Additionally, small molecules that bind DNA lack sequence specificity and, therefore, have significant off-target toxicity and effects [30,54]. Mithramycin is not significantly different. Despite being a potent Sp1 inhibitor, toxicity is considerable with the standard formulation.

**Figure 3:**
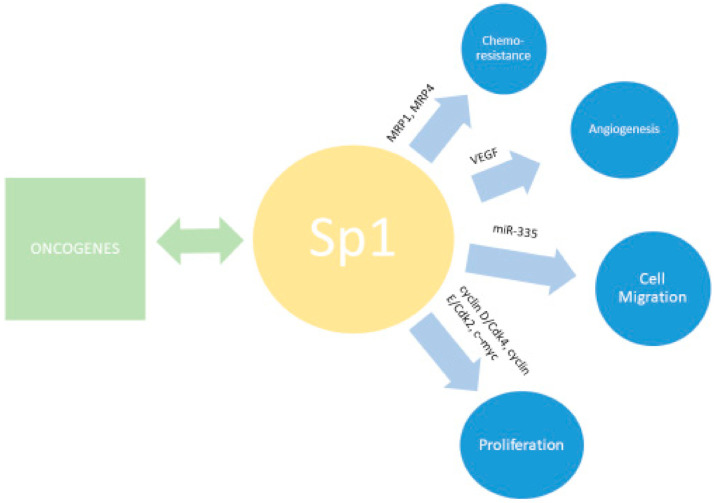
Sp1 is involved in a number of downstream effects related to tumor progression.

Due to the central role of Sp1 in cell cycle regulation and cell growth, aberrations in its expression, specifically upregulation, have been linked to tumorigenesis in a number of malignancies, including ovarian cancer (Figure 3). In ovarian cancer cells, Sp1 can be upregulated, activating numerous oncogenes and leading to increased cell migration, angiogenesis, and decreased apoptosis [42,55]. CD147, an immunoglobin that is overexpressed in ovarian cancer and noted to be an independent prognostic factor, phosphorylates Sp1, and forms a positive feedback loop in vitro [56]. Additional studies demonstrated that upregulation of Sp1 increased expression of VEGF and subsequent angiogenesis and migration of SKOV3-T ovarian cell lines in vivo and in vitro [57]. Hepatitis B X-interacting protein (HBXIP), an oncoprotein overexpressed in ovarian cancer, was shown to promote cell migration via induced Sp1-mediated transcription [58]. Sp1 was also shown to suppress microRNA 335, for which low expression has been associated with poor prognosis in ovarian cancer and enhanced ovarian cancer cell migration in vivo [59]. Interestingly, targeting Sp1 has demonstrated prior success in cancer cell lines. Tolfenamic acid, a Sp1 inhibitor, was strongly synergistic with cisplatin in ES2 and OVCAR-3 cell lines in vitro [60]. In a large cohort of patients with ovarian cancer, Knappskog and colleagues demonstrated that MDM2 promoter polymorphisms resulted in decreased SP1 promoter binding with reduced SP1 expression and a resulting decrease in ovarian cancer risk [61], suggesting Sp1 inhibition as a potential therapeutic target in ovarian cancer [62].

Sp1 has also been implicated in chemotherapy resistance [63]. In a study utilizing platinum-resistant SKOV3 cells, SP1 was upregulated and involved in the transcription of drug resistance proteins, MRP1, and MRP4 [63]. Increased expression of SP1 in clear cell ovarian cancer lines reduces sensitivity to SN-38, an irinotecan derivative, by upregulating the expression of Heparin-binding epidermal growth factor-like growth factor (HB-EGF) in vitro [64]. Long non-coding RNA ZFAS1 was demonstrated to be overexpressed in epithelial ovarian cancer and was associated with platinum and taxol chemoresistance via targeting a MiR-150-5p/SP1 axis [65]. In an analysis of the National Center for Biotechnology Information Gene Expression DatabaseGEO database, Sp1 was found to be overexpressed in platinum-resistant ovarian cancer patients [63]. Since Sp1-driven transcription plays an integral role in the pathogenesis of ovarian cancer and platinum-resistance mechanisms [38,42,66], and mithramycin has demonstrated a potent ability to inhibit Sp1, mithramycin or its more selective, safer analogs could be a potential strategy to overcome platinum resistance.

Further investigation of ovarian cancer stem cells using high-throughput screening identified mithramycin as a drug of interest [67]. Ovarian cancer stem cells have self-renewal capacity, resistance to numerous chemotherapy agents, and the ability to differentiate into different lineages. Of 825 compounds screened, mithramycin was noted to be a potent inhibitor of ovarian cancer stem cell proliferation [67]. In the same study, mithramycin was one of four FDA-approved agents that demonstrated additional inhibition of OVCAR-3 cell proliferation [67]. OVCAR-3 is a cisplatin-resistant ovarian cell line that is widely used as an in vitro model for platinum resistance in ovarian cancer [68,69]. Interestingly, OVCAR-3 is also resistant to doxorubicin, another chemotherapy agent derived from *Streptomyces* with a chemically distinct structure and a intercalative mode of DNA binding. Additional research in the early 2010s indicated a potentially significant role for mithramycin or a mithramycin-analog in the treatment of ovarian cancer, with promising anti-tumor effects demonstrated in vitro and in vivo [41]. Mithramycin analogs demonstrated inhibition of Sp1 dependent transcription in cell culture lines [37] and in human ovarian cancer xenografts [41]. Mithramycin and additional analogs demonstrated rapid intracellular accumulation in OVCAR-3 cell lines [38]. The IC_50_ values of mithramycin and related compounds were in the low-nanomolar range in OVCAR-3 cell cultures, induce cell arrest in G1 [70]. This finding raises the possibility of mithramycin or its analog as a potential treatment for chemotherapy-resistant ovarian cancer, particularly platinum-resistant cancer.

Additional studies in other malignancies have opened similar prospects for the treatment of chemoresistant lines. Mithramycin was observed to inhibit expression of ABCB8, a subunit of a potassium channel responsible for multi-drug resistance, in melanoma cells and sensitized chemotherapy-resistant breast cancer stem cells to doxorubicin via Sp1 inhibition [71,72]. Synergistic combination therapies could potentially circumvent the potential toxicity of mithramycin by employing lower doses of this agent in combination with additional chemotherapy agents. There is a paucity of data on synergistic effects of mithramycin in combination with other chemotherapeutic agents for the treatment of ovarian cancer, although there are some data in other cell lines. In a parallel screening of FDA drugs, mithramycin was noted to be a profound sensitizer for TRAIL-induced apoptosis in pancreatic cancer [73]. Additionally, mithramycin has been noted in vitro to have synergy with vorinostat in Sezary T lymphoma [74], bortezomib in multiple myeloma [75], cabazitaxel in drug-resistant acute B cell lymphoblastic leukemia [76], and betulinic acid and bevacizumab in pancreatic cancer [77,78]. Mithramycin was noted to have a synergistic effect with nutlin-3, a p53-MDM2 inhibitor, in an endometrial cancer cell line (HHUA) [44]. Also, the same study demonstrated significant in vitro and in vivo efficacy against ovarian cancer cell lines, including xenografts and intraperitoneal injections of mithramycin, albeit at considerably high doses (600 μg/kg/day) [76].

## 7. Development of Mithramycin Analogues

Whilst mithramycin has significant anticancer activity, severe toxicity precludes its use. This issue has prompted the development of novel and more selective mithramycin derivatives. The analogs contain the tricyclic chromophore of mithramycin, which preserves the DNA binding function while differing in the nature of the 3-side chain or the identity of the sugars. The varying side chains can be optimized to interact selectively with specific transcription factors and modulate the toxicity and efficacy of the analogs. [79]. Genetic approaches to alter the mithramycin biosynthesis pathway resulted in the synthesis of “mithralogs”, with altered pharmacological and biochemical properties [37]. Mithramycin analogs are synthesized by semisynthetic techniques using a mutant strain of a bacterial producer that generates a mithramycin precursor or a shunt product, which is then elaborated by standard chemical synthesis [39]. These mithramycin derivatives demonstrate promise with retained ability to inhibit Sp-1 transcription and tumorigenesis while reducing toxicity compared to unaltered mithramycin [31,38,40,41,80,81,82]. Most recently, mithramycin SA analogs, with 3-side chain modifications, showed highly improved selectivity of inhibition of Ewing sarcoma and TMPRSS2-ERG expressing prostate cancer cells over mithramycin [83]. Similarly, mithramycin 2′-oximes displayed improved selectivity against Ewing sarcoma cells in vitro as well as a better pharmacokinetic PK profile and somewhat superior efficacy in mouse Ewing sarcoma xenografts, relative to those of mithramycin [84].

One of the most promising mithralogs, demycarosyl-3D-betat-D-digitoxosyl-mithramycin SK (MTM-DIG-MSK or EC-8042), has demonstrated retained anti-tumor properties, including in vitro ovarian cancer lines, but reduced in vitro toxicity in fibroblasts and mononuclear blood cells, compared to mithramycin [37,40,70,82]. Mithramycin and EC-8042 reduced the viability and invasiveness of malignant melanoma cell lines [85]. EC-8042 inhibited SP1 transcription in sarcoma cell lines with the induction of cell cycle arrest and apoptosis, and interestingly was not a substrate for several drug resistance efflux pumps [86]. Compared to mithramycin SK, another mithramycin analog, EC-8042 had greater anti-transcriptional effects in colon carcinoma cell lines [82]. Additionally, in a prostate cancer mouse model, mithramycin analogs were 4-32x better tolerated than standard mithramycin, and exhibited considerable anti-tumor effects against xenografts without evidence of systemic toxicity [87]. There is also some evidence to suggest a synergistic effect with other chemotherapeutic agents in some cancer cell lines. EC-8042 potentiated the effect of docetaxel in triple-negative breast cancer cells in mouse xenograft models. There was no synergy with carboplatin in that setting [88].

Mithramycin analogs also demonstrated efficacy in pre-clinical ovarian studies. EC-8042 displayed significant anti-tumor activity against ovarian cancer cell lines with significantly lower toxicity to fibroblasts and peripheral blood cells compared to mithramycin; while strongly inhibiting Sp1 transcription and reduction in several genes implicated in tumorigenesis including VEGF, BRCA2, cMyc, and src [38,70]. In one of the most detailed studies involving ovarian cancer and MTM analogs, MTM-SDK and MTM-SK, demonstrated considerable inhibition of Sp1 dependent transcription in vitro. Additionally, a mouse model demonstrated significant growth inhibition of subcutaneous ovarian xenografts for both compounds. In an orthotopic xenograft model, MTM-SDK demonstrated a significant increase in the median time of abdominal distension/ascites and median survival. Importantly, the compound was well tolerated without signs of toxicity in the mouse model [41].

While the recent clinical trials involving mithramycin were underwhelming, there have been no recent trials investigating mithramycin analogs or combination therapies with other agents. Novel drug delivery vehicles for mithramycin have been explored with reported success. It has been demonstrated that polymeric nanoparticles of mithramycin can be formulated by techniques suitable for amphiphilic compounds [89]. Via a unique delivery approach, mithramycin loaded nanoparticles have demonstrated considerable efficacy against pancreatic cancer xenografts [90]. Mithramycin analogs MTM-SK and MTM-SDK were also placed in polymer micelles in order to increase bioavailability and drug targeting, with improved cytotoxicity compared to free drug in non-small cell lung cancer lines [91]. Of note, there have been no investigations of such novel drug delivery mechanisms for mithramycin or analogs in ovarian cancer.

## 8. Conclusions

Transcription factor targeting remains a challenging oncological goal. There is ample evidence to suggest that mithramycin is a potent Sp-1 inhibitor and has demonstrated anticancer effects across a wide range of malignancies, including ovarian, in pre-clinical models. The widespread utility of mithramycin continues to be limited by a narrow therapeutic window and profound hepatotoxicity. However, recent developments in drug delivery and the generation of novel analogs are particularly promising. Mithramycin analogs have retained anti-tumor effects in vitro, and limited toxicity has been observed in animal models while gaining selectivity and improved PK properties. With recent advances and data demonstrating a profound role of intraperitoneal chemotherapy in ovarian cancer therapy, a potential role for mithramycin is an uninvestigated and intriguing avenue of inquiry [92]. One of the most intriguing possibilities is the utility of mithramycin or an analog for the treatment of platinum-resistant ovarian cancer, an unmet therapeutic need. There have been several studies demonstrating the in vitro efficacy of mithramycin against platinum-resistant ovarian cancer, but limited investigation with in vivo models. Further studies utilizing pre-clinical animal models are warranted to further evaluate potential toxicities prior to consideration in human subjects. There remains considerable potential for mithramycin in relation to ovarian cancer therapy, and there are a number of unexplored areas of investigation.
